# Exploring Amino Acid Auxotrophy in *Bifidobacterium bifidum* PRL2010

**DOI:** 10.3389/fmicb.2015.01331

**Published:** 2015-11-24

**Authors:** Chiara Ferrario, Sabrina Duranti, Christian Milani, Leonardo Mancabelli, Gabriele A. Lugli, Francesca Turroni, Marta Mangifesta, Alice Viappiani, Maria C. Ossiprandi, Douwe van Sinderen, Marco Ventura

**Affiliations:** ^1^Laboratory of Probiogenomics, Department of Life Sciences, University of ParmaParma, Italy; ^2^GenProbio Ltd.Parma, Italy; ^3^Department of Medical-Veterinary Science, University of ParmaParma, Italy; ^4^APC Microbiome Institute and School of Microbiology, University College Cork, National University of IrelandCork, Ireland

**Keywords:** bifidobacteria, chemically defined medium, genomics, microbiota

## Abstract

The acquisition and assimilation strategies followed by members of the infant gut microbiota to retrieve nitrogen from the gut lumen are still largely unknown. In particular, no information on these metabolic processes is available regarding bifidobacteria, which are among the first microbial colonizers of the human intestine. Here, evaluation of amino acid auxotrophy and prototrophy of *Bifidobacterium bifidum*, with particular emphasis on *B. bifidum* strain PRL2010 (LMG S-28692), revealed a putative auxotrophy for cysteine. In addition, we hypothesized that cysteine plays a role in the oxidative stress response in *B. bifidum*. The use of glutathione as an alternative reduced sulfur compound did not alleviate cysteine auxotrophy of this strain, though it was shown to stimulate expression of the genes involved in cysteine biosynthesis, reminiscent of oxidative stress response. When PRL2010 was grown on a medium containing complex substrates, such as whey proteins or casein hydrolysate, we noticed a distinct growth-promoting effect of these compounds. Transcriptional analysis involving *B. bifidum* PRL2010 cultivated on whey proteins or casein hydrolysate revealed that the biosynthetic pathways for cysteine and methionine are modulated by the presence of casein hydrolysate. Such findings support the notion that certain complex substrates may act as potential prebiotics for bifidobacteria in their ecological niche.

## Introduction

The gut lumen contains a very complex mixture of compounds from alimentary and endogenous origins together with living microorganisms. The intestinal microbiota is metabolically active and plays a significant role in host physiology and metabolism (Hamer et al., 2012). The ability to metabolize peptides and amino acids is shared by a large number of bacteria ranging from saccharolytic bacteria to obligate amino acid fermenters present in gut microbiota (Davila et al., 2013). Peptides are the preferred substrates over free amino acids for many colonic bacteria, probably due to kinetic advantages of peptide uptake systems. Moreover, nitrogen source, such as amino acids, are fermented to short-chain fatty acids and organic acids, representing energy fuel for the colonic mucosa (Davila et al., 2013).

Milk proteins and peptides such as lactoferrin, lactoperoxidase, and lysozyme are reported to provide a non-immune defense against microbial infections (Schanbacher et al., 1997). In addition, they are known to stimulate growth of several members of the human infant microbiota such *Lactobacillus* and *Bifidobacterium* (Liepke et al., 2002; McCann et al., 2006). In this latter ecological context, the bacterial population is dominated by bifidobacteria, which remain a prominent component of the gut microbiota until weaning (Turroni et al., 2012a; Duranti et al., 2015; Underwood et al., 2015). Member of the genus *Bifidobacterium* are anaerobic microorganisms, typically resident in the gastro intestinal tract of mammals and insects (Lugli et al., 2014), where they are known to interact with their hosts using various genetic strategies (O’Connell Motherway et al., 2011; Fanning et al., 2012; Ventura et al., 2012; Turroni et al., 2014).

Among host-derived nutrients, milk proteins significantly influence the composition of the gut microbiota, supplying these microorganisms with nitrogen and amino acids (Liepke et al., 2002). Enhancement of (bifido)bacterial growth is frequently associated with milk proteins and the peptides that arise from the hydrolysis of these proteins (Nagpal et al., 2011; Lonnerdal, 2013).

Compared to carbon metabolism, for which a large body of scientific data is available (Pokusaeva et al., 2011; Marcobal et al., 2013), only very limited knowledge is available on the acquisition and assimilation processes that are used by members of the infant gut microbiota to retrieve nitrogen from the gut lumen (Liepke et al., 2002). For Gram positive bacteria, nitrogen metabolism has been investigated in *Lactobacillus delbrueckii* subsp. *bulgaricus* (Liu et al., 2012), *Lactobacillus rhamnosus* (Lebeer et al., 2007) and *Bacillus* sp. (Fisher, 1999; Even et al., 2006).

**FIGURE 1:**
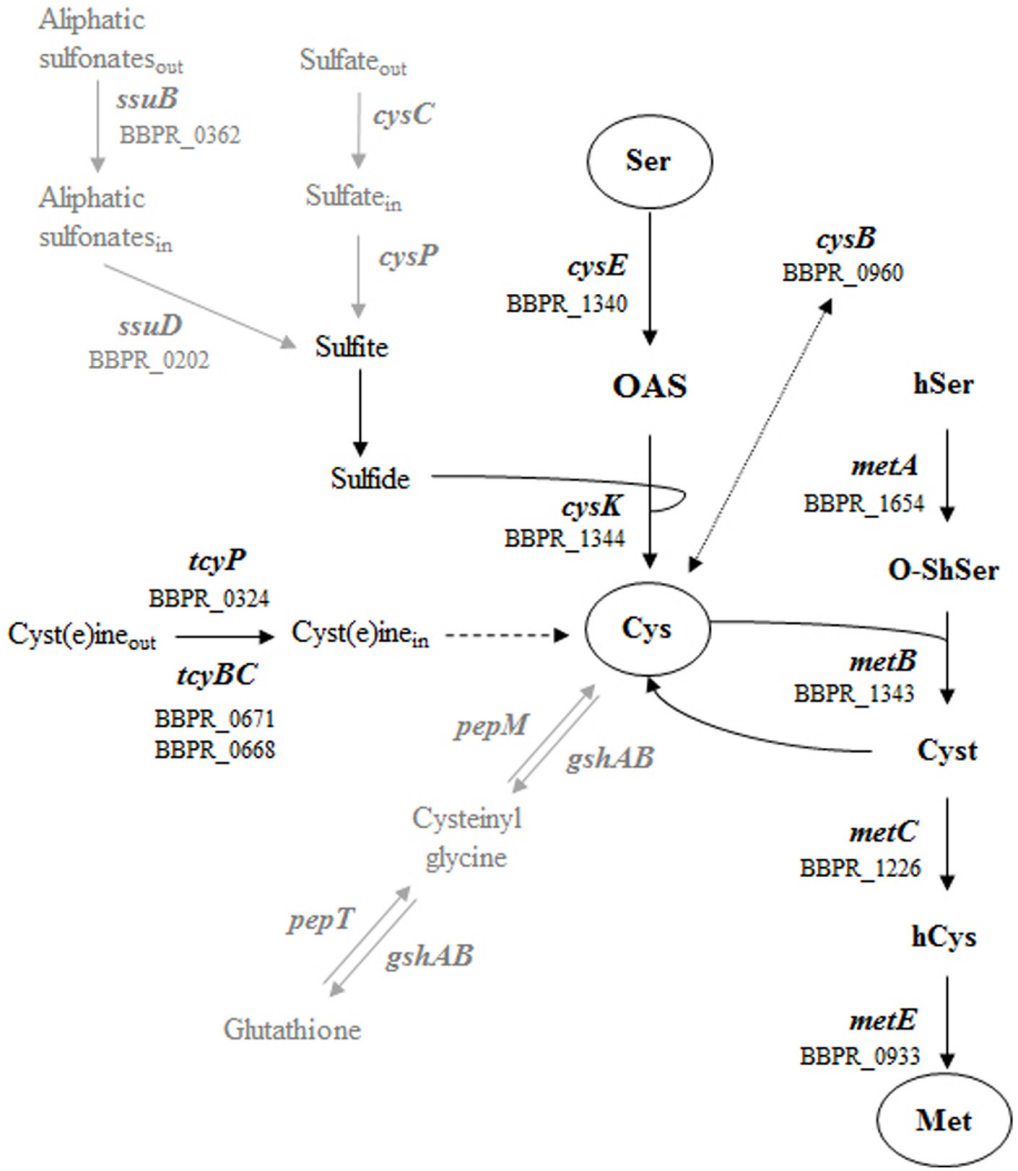
**Schematic representation of the metabolic pathways for sulfur amino acid in Gram positive bacteria.** The different ORFs of *Bifidobacterium bifidum* PRL2010 encoding the predicted enzymes are indicated. The metabolic steps present in Gram positive bacteria but absent in PRL2010 (sulfate assimilation and glutathione synthesis) are indicated by gray arrows. Molecule involved are reported as follow: serine Ser, *O*-acetyl-L-serine OAS, cysteine Cys, homoserine hSer, *O*-succinylhomoserine *O*-ShSer, cystathionine Cyst, homocysteine hCys and methionine Met.

Recently, specific interest has been directed toward sulfur-containing amino acids and global control of cysteine and methionine metabolism in both Gram positive and negative bacteria, such as *Lactococcus lactis*, *Salmonella* sp., *Vibrio fischeri* and *Clostridium perfringens* (Fernandez et al., 2002; Andre et al., 2010; Alvarez et al., 2015; Singh et al., 2015). Cysteine biosynthesis is the key mechanism by which inorganic sulfur is reduced and incorporated into organic compounds (Kredich, 1992), where it plays an essential role in the formation of the catalytic sites of several enzymes, or protein folding and assembly via the formation of disulfide bonds (Mihara and Esaki, 2002). Sulfur-containing compounds that are used for the synthesis of cysteine and methionine are transported into the bacterial cell through different mechanisms: the first involves sulfate permease related to inorganic phosphate transporters (CysC) and then the reduction of sulfate to sulfide (Mansilla and de Mendoza, 2000) (**Figure 1**). The second involves aliphatic sulfonate ATP-binding cassette (ABC) transporters (SsuBD) (van der Ploeg et al., 1998) and the subsequent conversion into sulfide by an FMNH monooxygenase (**Figure 1**). The following reaction of sulfide with *O*-acetyl-L-serine (OAS) results in cysteine synthesis by the action of an *O*-acetylserine thiol-lyase (Bogicevic et al., 2012). Alternatively, cysteine can be directly transported inside the cell by symporter proteins (TcyBCP) (Burguiere et al., 2004). Methionine biosynthesis is closely linked to cysteine production by the action of serine acetyltransferase, which uses cysteine and an *O*-acetylhomoserine to generate cystathionine, where the latter compound is then converted to homocysteine and methionine (Fernandez et al., 2002) (**Figure 1**).

In this study, in order to understand the role of the bifidobacterial population in the utilization of nitrogen available in the human gut, we evaluated the amino acid metabolism of the infant stool isolate *B. bifidum* PRL2010 (LMG S-28692), a bifidobacterial prototype for analysis of interactions between microbes and the intestinal mucosa (Turroni et al., 2013), by coupling physiological data on a chemically defined medium (CDM) with transcriptional analysis. Specific emphasis was placed on sulfur amino acids/metabolism of PRL2010 since these amino acids are particularly important for the bacterial cells of gut commensals in coping against gut related stresses (e.g., oxidative stress) (Even et al., 2006).

Furthermore, PRL2010 metabolism of complex substrates from milk such as casein hydrolysate and whey proteins was investigated.

## Materials and Methods

### Bacterial Strains and DNA Extraction

Bifidobacterial strains used in this study are reported in **Table 1**. Strains were grown anaerobically in de Man, Rogosa, Sharpe (MRS) medium (Scharlau, Spain), which was supplemented with 0.05% L-cysteine-HCl and incubated at 37°C for 16 h. Anaerobic conditions were achieved by the use of an anaerobic cabinet (Ruskin), in which the atmosphere consisted of 10% CO_2_, 80% N_2_, and 10% H_2_.

**Table 1:** Bifidobacterial strains used in this study.

Bacteria	Strains^a^	Genome accession numbers
*B. actinocoloniiforme*	DSM 22766	JGYK00000000
*B. adolescentis*	ATCC 15703	AP009256.1
*B. angulatum*	LMG 11039	JGYL00000000
*B. animalis* subsp. *animalis*	LMG 10508	JGYM00000000
*B. animalis* subsp. *lactis*	DSM 10140	CP001606.1
*B. asteroides*	LMG 10735 (PRL2011)	CP003325.1
*B. biavatii*	DSM 23969	JGYN00000000
*B. bifidum*	LMG 11041	JGYO00000000
*B. bifidum*	PRL2010	CP001840
*B. bifidum*	85B	JSDU00000000
*B. bifidum*	324B	JSDT00000000
*B. bifidum*	156B	JSDS00000000
*B. bifidum*	LMG 11583	JSDZ00000000
*B. bifidum*	LMG 11582	JSDY00000000
*B. bifidum*	LMG 13200	JSEB00000000
*B. bifidum*	LMG 13195	JSEA00000000
*B. bohemicum*	DSM 22767	JGYP00000000
*B. bombi*	DSM 19703	ATLK00000000
*B. boum*	LMG 10736	JGYQ00000000
*B. breve*	LMG 13208	JGYR00000000
*B. callitrichos*	DSM 23973	JGYS00000000
*B. catenulatum*	LMG 11043	JGYT00000000
*B. choerinum*	LMG 10510	JGYU00000000
*B. coryneforme*	LMG 18911	CP007287
*B. crudilactis*	LMG 23609	JHAL00000000
*B. cuniculi*	LMG 10738	JGYV00000000
*B. dentium*	LMG 11405 (Bd1)	CP001750.1
*B. gallicum*	LMG 11596	JGYW00000000
*B. gallinarum*	LMG 11586	JGYX00000000
*B. indicum*	LMG 11587	CP006018
*B. kashiwanohense*	DSM 21854	JGYY00000000
*B. longum* subsp. *infantis*	ATCC 15697	AP010889.1
*B. longum* subsp. *longum*	LMG 13197	JGYZ00000000
*B. longum* subsp. *suis*	LMG 21814	JGZA00000000
*B. magnum*	LMG 11591	JGZB00000000
*B. merycicum*	LMG 11341	JGZC00000000
*B. minimum*	LMG 11592	JGZD00000000
*B. mongoliense*	DSM 21395	JGZE00000000
*B. moukalabense*	DSM 27321	AZMV00000000.1
*B. pseudocatenulatum*	LMG 10505	JGZF00000000
*B. pseudolongum* subsp. *globosum*	LMG 11596	JGZG00000000
*B. pseudolongum* subsp. *pseudolongum*	LMG 11571	JGZH00000000
*B. psychraerophilum*	LMG 21775	JGZI00000000
*B. pullorum*	LMG 21816	JGZJ00000000
*B. reuteri*	DSM 23975	JGZK00000000
*B. ruminantium*	LMG 21811	JGZL00000000
*B. saeculare*	LMG 14934	JGZM00000000
*B. saguini*	DSM 23967	JGZN00000000
*B. scardovii*	LMG 21589	JGZO00000000
*B. stellenboschense*	DSM 23968	JGZP00000000
*B. stercoris*	DSM 24849	JGZQ00000000
*B. subtile*	LMG 11597	JGZR00000000
*B. thermacidophilum* subsp. *porcinum*	LMG 21689	JGZS00000000
*B. thermacidophilum* subsp. *thermacidophilum*	LMG 21395	JGZT00000000
*B. thermophilum*	JCM 1207	JGZV00000000
*B. tsurumiense*	JCM 13495	JGZU00000000


***^a^**ATCC, American Type Culture Collection, USA. LMG, Belgian Co-ordinated Collection of Microorganisms-Bacterial Collection, Belgium. DSM, German Collection of Microorganism and Cell Cultures, Germany. JCM, Japan Collection of Microorganisms, Japan.*

### *Bifidobacterium* CDM Development

For amino acid auxotrophy and prototrophy tests, a CDM was employed based on a previously described formulation (Petry et al., 2000; Cronin et al., 2012) for *Lactobacillus* and *Bifidobacterium*, with some modifications. Briefly, to the already reported CDM, 50 mg/l of guanine and 4.0 mg/l of thiamine was added. Several simple sugars were screened including glucose, fructose, galactose, lactose, ribose, xylose, fucose, mannose, and rhamnose. All carbohydrates were added at 2% (w/w). The medium was sterilized by filtration (0.22 μm). When the CDM was prepared without amino acids it is termed basal CDM (bCDM). All components of CDM were purchased from Sigma (USA).

### Amino Acid and Nitrogen Growth Assay

Cell growth on CDM was monitored by measuring the optical density of cultures at 600 nm (OD 600) using a plate reader (Biotek, Winooski, VT, USA). The plate reader was run in discontinuous mode, with absorbance readings performed after 24 h of incubation and preceded by 30 s of shaking at medium speed. Bacteria were cultivated in the wells of a 96-well microtiter plate, with each well containing a different amino acid, and incubated in an anaerobic cabinet.

For all growth tests, cells were recovered from an overnight MRS broth culture, centrifuged at 3000 rpm for 5 min in anaerobiosis, and washed with bCDM to remove protein and sugar residues. In each of the 96-wells of the microplate, 135 μl of medium was inoculated with 15 μl of washed cells diluted to OD 1.0 with bCDM, obtaining a final inoculum OD of 0.1. Wells were covered with 30 μl of sterile mineral oil in order to maintain anaerobic conditions. Cultures were grown in biologically independent triplicates and the resulting growth data were expressed as the means from these replicates.

Once it had been established that CDM supports bifidobacterial growth, amino acid assays were performed using CDM in which individual amino acids were omitted or with bCDM supplemented with 0.2 g/l of a particular amino acid. To understand amino acid metabolism and in particular the metabolism of sulfur-containing amino acids derived from complex substrates, bCDM was supplemented with 2–0.5% (w/w) of whey protein or casein hydrolysate (Sigma). To test the influence of reduced sulfur substrate instead cysteine to PRL2010 growth, 5 mM of reduced glutathione (Sigma) was added to bCDM formulation without any other amino acid (bCDM + Glut). To evaluate the utilization of source of sulfur and nitrogen available in the gut environment, 0.5 g/l of taurine was added to bCDM.

### Identification of Genes Involved in Sulfur-containing Amino Acid Metabolism

The identification of genes involved in cysteine and methionine metabolism in PRL2010 and other *B. bifidum* strains was performed by using the BLASTP program (Gish and States, 1993). For the BLAST search, previously identified genes involved in sulfur metabolism of lactic acid bacteria were used (Liu et al., 2012). Twenty bp oligonucleotides for RT-qPCR experiments were manually designed on identified putative genes to obtain amplicons with a size ranging from 150 to 200 bp. Primers were checked with Primer Blast (Ye et al., 2012) and listed in **Table 2**.

**Table 2:** Primers used for RT-qPCR experiments.

Target	ORF	Primer Fw 5′-3′		Primer Rv 5′-3′		Size (bp)
*cysE*	BBPR_1340	cysE-fw	CGCGACCATGCGCGACTACC	cysE-rv	GAGGATGCGCTCGTGTCCGC	187
*cysK*	BBPR_1344	cysK-fw	CGAACCAGTACGACAACCCC	cysK-rv	GATGGAGCCTTCCGGATCGG	203
*cysB*	BBPR_0960	cysB-fw	GACGACCTCAAGCCGTTCCC	cysB-rv	GTCGCCGTTGTCGATGCCGG	189
*metB*	BBPR_1343	metB-fw	GGAGCCCGACCCGACCACCG	metB-rv	CAGCAGCACGTCAATCGCGG	214
*metC*	BBPR_1226	metC-fw	CATGGGTGTGGGAAGCGAGG	metC-rv	TCGATGTCCCAGTTGTGCCG	189
*metE*	BBPR_0933	metE-fw	GATGCTGGACACCGCGATCC	metE-rv	GGCGGATCTCGGTGCTCTCC	206
*metA*	BBPR_1654	metA-fw	GTTCGCTCTCGGCCATTGGG	metA-rv	CGGCGTGGTCTGATACACCC	205
*rpoB^a^*		BBP-rpo-for	GTGCAGACCGACAGCTTCGAC	BBP-rpo-rev	GAGATCTCGTTGAAGAACTCGTC	
*ldh^a^*		BBP-ldh-for	CACCATGAACAGGAACAAAGTTG	BBP-ldh-rev	GAATGATCGATGAGTACGAGCTC	
*atpD^a^*		BBP-atp-uni	CAGAGCCGATCAATGGACGTG	BBP-atp-rev	GTGCTGCTCGACCTCAAGCGTGAT	


**^a^*Turroni et al. (2011).*

### RNA Isolation, Reverse Transcription and RT-qPCR

Total RNA was isolated from PRL2010 cultures grown in CDM, bCDM supplemented with cysteine, or bCDM supplemented with cysteine and whey protein or casein hydrolysate (2% w/w). PRL2010 cells grown in MRS was used as a control condition. Cultures were grown in biologically independent triplicates. Cells were harvested by centrifugation step at 4000 ×*g* for 5′ at 4°C when cells had reached late exponential phase (OD values of 0.8–1.0, except for bCDM supplemented with glutathione where cells were harvested at OD 0.35). Cell pellets were resuspended in 500 μl of RNAprotect reagent (Qiagen, UK) and mechanically lysed by inclusion of 0.1 mm zirconium–silica beads (Biospec Products, Bartlesville, OK, USA) and by subjecting the sample to three 2 min pulses at maximum speed in a bead beater (Biospec Products, Bartlesville, OK, USA) with intervals of 3 min on ice. RNA was extracted with the RNeasy mini kit (Qiagen) as reported in the manufacturer’s instructions. Quality and integrity of the RNA was checked by Tape station 2200 (Agilent Technologies, USA) analysis and only samples displaying a RIN value above seven were used. RNA concentration and purity was then determined with a Picodrop microlitre Spectrophotometer (Picodrop). Reverse transcription to cDNA was performed with the iScript Select cDNA synthesis kit (Biorad) using the following thermal cycle: 5 min at 25°C, 30 min at 42°C, 10 min at 45°C, 10 min at 50°C and 5 min at 85°C.

The mRNA expression levels of these genes were analyzed with SYBR green technology in quantitative real-time PCR (qRT-PCR) using SoFast EvaGreen Supermix (Biorad) on a Bio-Rad CFX96 system according to the manufacturer’s instructions. Quantitative PCR was carried out according to the following cycle: initial hold at 96°C for 30 s and then 40 cycles at 96°C for 2 s and 60°C for 5 s. Gene expression was normalized relative to a housekeeping genes as previously described (Turroni et al., 2011) and reported in **Table 2**. The amount of template cDNA used for each sample was 12.5 ng.

### Statistical Analyses

Statistical significance between means was analyzed using the two way ANOVA. Statistically different means were determined using the Bonferroni *post hoc* test at 5% (*P*-value < 0.05). Values are expressed as the means ± standard errors from three experiments. Statistical calculations were performed using the software program GraphPad Prism 5 (La Jolla, CA, USA).

## Results

### Development of a CDM for *B. bifidum* PRL2010

We modified the previously described CDM formulations (Petry et al., 2000; Cronin et al., 2012) based on the nutrient requirements of *B. bifidum* PRL2010. Several growth attempts on CDM minimal modifications (Petry et al., 2000; Cronin et al., 2012), i.e., where various compounds were omitted one after the other, allowed the identification of a number of components that were either essential or non-essential for growth of PRL2010 cells. Notably, folic acid and pyridoxal were eliminated from CDM_PRL2010_ composition, while guanine and thiamine were supplemented. When testing different sugars it was observed that PRL2010 exhibits the best growth performance with lactose, consistent with previous studies (Turroni et al., 2010, 2012b), and this sugar was therefore used for CDM_PRL2010_ formulation.

### Evaluation of Amino Acids Auxotrophy and Prototrophy of PRL2010

**FIGURE 2:**
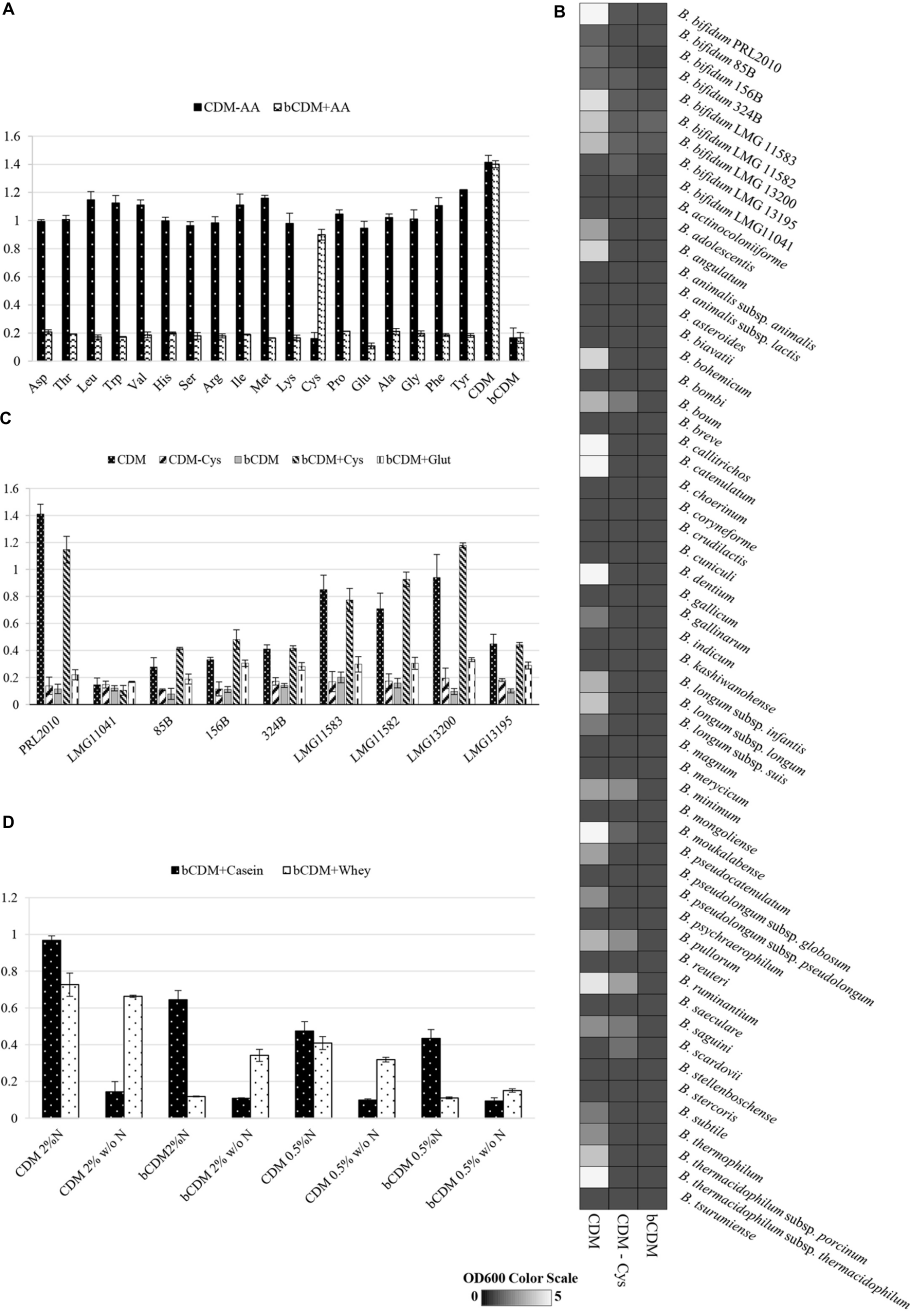
**Growth of *B. bifidum* strains**. Growth was measured as the optical density of the medium at 600 nm (OD600). Cultures were grown in triplicates. **(A)** Reports the growth of *B. bifidum* PRL2010 in CDM_PRL2010_. In these tests, one amino acid at time was removed (CDM-AA) or supplied (bCDM + AA) to the medium. Amino acids are reported in the horizontal axis as follows: aspartic acid Asp, threonine Thr, leucine Leu, tryptophan Trp, valine Val, histidine His, serine Ser, arginine Arg, isoleucine Iso, methionine Met, lysine Lys, cysteine Cys, proline Pro, glutamine Gln, alanine Ala, glycine Gly, phenylalanine Phe and tyrosine Tyr. **(B)** Shows an heat map representing the growth performance of all of the type strains of the currently recognized 48 (sub)species belonging to the genus *Bifidobacterium* on CDM_PRL2010_, CDM-Cys_PRL2010_ and bCDM_PRL2010_. The different shading represents the optical density reached by the various cultures. **(C)** Displays the growth of *B. bifidum* strains LMG11041, 85B, 156B, 324B, LMG11583, LMG11582 LMG13200 and LMG13195 in comparison with PRL2010 in CDM_PRL2010_, CDM_PRL2010_ without cysteine (CDM – Cys), basal CDM_PRL2010_ (bCDM), basal CDM_PRL2010_ with cysteine (bCDM + Cys) and basal CDM_PRL2010_ with glutathione (bCDM + Glut). **(D)** Illustrates the growth of *B. bifidum* PRL2010 in CDM_PRL2010_ supplemented with complex substrates like whey proteins or casein hydrolysate. For both substrates two concentration were tested, 0.5 and 2% (wt/wt). For every concentration was evaluated the presence of amino acid (CDM or bCDM) or other nitrogen sources (N or w/o N).

When PRL2010 cells were cultivated on CDM_PRL2010_, they exhibited reduced growth (OD600 value of 1.21 ± 0.3) compared to that observed when grown on a nutrient-rich medium such as MRS (OD600 value of 2.9 ± 0.2). In order to assess PRL2010 amino acid prototrophy/auxotrophy, growth experiments were performed using CDM_PRL2010_ where an individual amino acid had been omitted at time, and bCDM_PRL2010_ medium with the inclusion of one amino acid at time. The achieved growth yield was compared to that obtained for complete CDM_PRL2010_ or bCDM_PRL2010_ respectively (**Figure 2A**).

In both experiments, only when cysteine was removed or supplied to the medium a significant decrease or increase (ranging from four- to sixfold, *P* < 0.05) of the obtained growth yield was observed, respectively, suggesting that PRL2010 is auxotrophic for cysteine. Furthermore, PRL2010 seems unable to grow on sulfate as its sole sulfur source, such as when this strain is grown in bCDM_PRL2010_ (a medium that contains MnSO_4_, MgSO_4_, and FeSO_4_).

Another reducing compound, glutathione, was added to bCDM_PRL2010_ and only very limited growth was detected when PRL2010 cells were cultivated for 24 h (OD600 value of 0.31 ± 0.038). Furthermore, we decided to investigate if taurine, which is a common nitrogen and sulfur sources present in the gut environment (Carbonero et al., 2012) influence the growth yields of PRL2010. However, we did achieved any significant grow (OD600 = 0.10 ± 0.01) of PRL2010 when taurine was used as the unique nitrogen and sulfur sources.

### Assessing Cysteine Auxotrophy of Members of the Genus *Bifidobacterium*

We further investigated the behavior of other strains belonging to the *B. bifidum* species (see **Table 1**), when cultivated under similar growth conditions (**Figure 2B**). These experiments showed that strains LMG11041, 156B, 85B, 324B, and LMG13195 were unable to grow on CDM_PRL2010_, (OD600 values ≤ 0.3) after 24 h of incubation (**Figure 2B**). The other *B. bifidum* strains investigated (i.e., LMG13200, LMG11582, and LMG11583) reached OD600 values of 0.7–0.9 and exhibited an identical auxotrophic behavior as PRL2010 for cysteine (**Figure 2B**).

Within the genus *Bifidobacterium*, the same auxotrophic behavior for cysteine appears to be widely distributed. In fact, of the currently recognized 48 (sub)species harboring the genus *Bifidobacterium*, only *B. boum* LMG10736, *B. minimum* LMG11592, *B. pullorum* LMG21816, *B. ruminantium* LMG21811, *B. saguini* DSM23967 and *B. scardovii* LMG21589 were shown to be able to grow in CDM_PRL2010_ without cysteine, though such strains reached OD600 values of just 0.5 ± 0.1 (**Figure 2C**). No bifidobacterial strain was able to grow in bCDM_PRL2010_.

### Sulfur Amino Acid Metabolism of *B. bifidum* PRL2010

A general prediction based on genomic data about nitrogen metabolism within the genus *Bifidobacterium* was previously reported by (Milani et al., 2014). The presence of genes involved in amino acid biosynthesis appears to be conserved among the seven phylogenetic groups of the genus *Bifidobacterium* (Lugli et al., 2014). However, the genes that are predicted to be involved in sulfur-containing amino acid metabolism were shown to be variably present within bifidobacterial genomes. In this context, an *in silico* analysis of the *B. bifidum* PRL2010 genome (Turroni et al., 2010) for putative genes involved in sulfur-containing amino acid transport did not reveal any positive match.

Aliphatic sulfonates can be used as alternative sulfur sources for the synthesis of cysteine (van der Ploeg et al., 1998). Bioinformatics analyses revealed the occurrence of two genes (BBPR_0202 and BBPR_0362) encoding two putative ABC-type permeases, in the chromosome of PRL2010. A low level of homology with genes involved in sulfonate transport (Even et al., 2006) was detected (**Supplementary Table S1**), possibly explaining why *B. bifidum* PRL2010 cells are unable to grow with sulfate as the only sulfur source (bCDM condition, see **Figure 2A**). Another mechanism to achieve sulfur from the environment is based on the intake of cysteine by symporter proteins. This type of symporter may participate in the uptake of cysteine (Vitreschak et al., 2008). In this context, a putative sodium dicarboxylate symporter gene (BBPR_0324) was identified in PRL2010 (see **Supplementary Table S1**). Moreover, two putative genes (BBPR_0668 and BBPR_0671) predicted to encode two carriers involved in glutamate transport system (GluA and GluD), exhibited 53 and 26% homology, respectively, with the genes that encode the L-cysteine uptake system of *B. subtilis* (**Supplementary Table S1**).

As mentioned above, *B. bifidum* PRL2010 cells were shown to be unable to grow in presence of reduced glutathione (and in the absence of cysteine). Such physiological findings are in agreement with *in silico* analyses of PRL2010 chromosome sequences. In fact, the *pepT* and *pepM* genes, which are constituting the pathway for degradation of this compound (Andre et al., 2010) to generate cysteine, are absent in PRL2010 genome. Furthermore, a homolog of the *gshAB* gene, which specifies the glutamate–cysteine ligase/glutathione synthase, is also absent in chromosome of PRL2010 (**Figure 1**).

**FIGURE 3:**
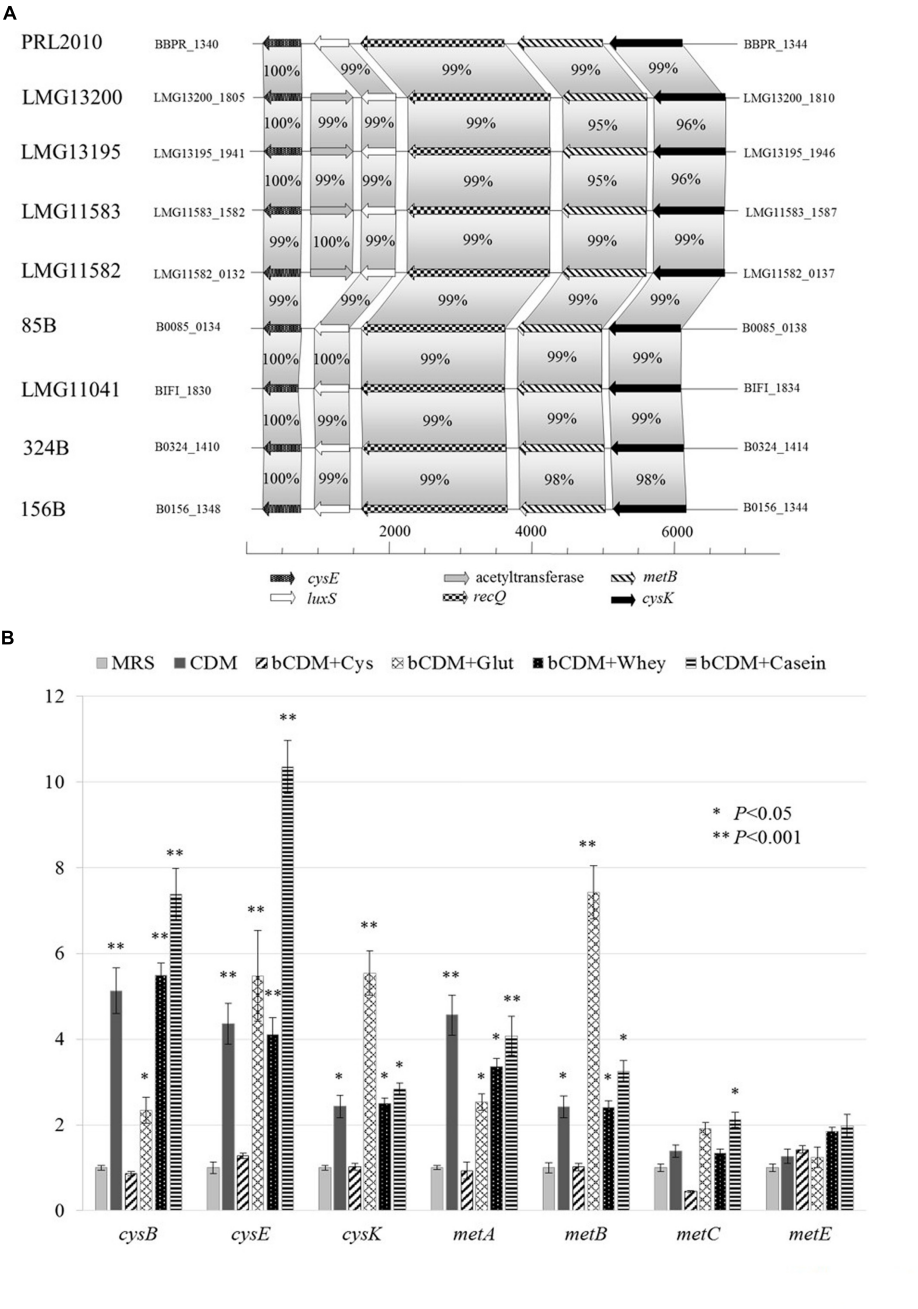
**Schematic representation of genes involved in cysteine and methionine metabolism in *B. bifidum* species and transcriptional analysis in *B. bifidum* PRL2010.** **(A)** Shows the genetic map of the predicted cysteine/methionine metabolism gene region identified in the genome of *B. bifidum* PRL2010 and compared with other *B. bifidum* strains. Each individual gene is represented by an arrow and is colored or marked according to the predicted function as indicated in the figure. **(B)** Reports the relative transcription levels of cysteine and methionine metabolism genes from *B. bifidum* PRL2010 upon cultivation in complete CDM_PRL2010_, bCDM_PRL2010_ supplemented with cysteine (bCDM + Cys), bCDM_PRL2010_ supplemented with glutathione (bCDM + Glut), bCDM_PRL2010_ with cysteine and 2% (wt/wt) whey protein (bCDM + Whey) and bCDM_PRL2010_ with cysteine and 2% (wt/wt) casein hydrolysate as analyzed by quantitative real-time PCR assays. The histograms indicate the relative amounts of the *cysE, cysK, cysB, metA, metE, metB*, and *metC* genes mRNAs for the specific samples. The *y* axis indicates the logarithmic fold induction of the investigated gene compared to the reference condition (MRS). The *x* axis represents the different gene tested. Asterisks indicate statistically significant differences compared to the control. The error bar for each column represent the standard deviation calculated from three replicates.

Genes predicted to be involved in the cysteine biosynthesis I/homocysteine degradation pathway and methionine biosynthesis I pathway were identified in PRL2010 (**Figure 3A**). *In silico* analyses of PRL2010 genome revealed the occurrence of the *cysE* (BBPR_1340) and *cysK* (BBPR_ 1344) genes, which encode the predicted serine acetyltransferase that transfers an acetyl group to serine, and the cysteine synthase, respectively (Liu et al., 2012) (**Figure 3A**). In the same genomic region, we also identified the *metB* gene (BBPR_1343) predicted to encode a cystathionine-γ-synthase, which is catalyzing the conversion of cysteine to cystathionine, as well as the *luxS* gene (BBPR_1341), encoding an *S*-ribosylhomocysteinase involved in the production of homocysteine, and the *recQ* gene (BBPR_1342) encoding an ATP-dependent DNA helicase. When the presence of these genes was investigated in the genomes of other *B. bifidum* strains (Duranti et al., 2015) included in this study, a high level of homology (higher than 98% at nucleotide level) was found. Furthermore, in the genome sequences of four *B. bifidum* strains, i.e., LMG13200, LMG13195, LMG11582, and LMG11583 (Duranti et al., 2015), an additional acetyltransferase-encoding gene was identified (**Figure 3A**).

Other genes such as the *cysB* gene (BBPR_0960), *metC* (BBPR_1226) and *metA* (BBPR_1654) that are predicted to be involved in cysteine and methionine metabolism (Fernandez et al., 2002; Liu et al., 2012) are scattered across the PRL2010 genome.

### Growth Evaluation in Complex Substrates

The effects of complex substrates, such as whey proteins or casein hydrolysate, on PRL2010 growth were tested and are reported in **Figure 2D**. Whey proteins and casein hydrolysate were dissolved in CDM_PRL2010_ and bCDM_PRL2010_ with or without other nitrogen sources at 0.5 or 2% concentration (wt/wt), respectively. Casein hydrolysate better supports PRL2010 growth in presence of nitrogen, compared to what was observed when this strain was cultivated on whey proteins. In CDM or bCDM without other nitrogen sources, PRL2010 cells seemed to metabolize whey proteins more efficiently as displayed by the higher OD 600 values that were reached (**Figure 2D**).

### Targeted Gene Expression Analyses of PRL2010 with Different Sulfur Substrate

Transcription of genes involved in sulfur metabolism, such as those of cysteine (*cysE, cysK* and *cysB*) and methionine metabolism (*met*A, *metE, metB*, and *metC*), were investigated using a qRT-PCR approach, the results of which are reported in **Figure 3B**.

When PRL2010 cells were cultivated in the complete CDM_PRL2010_, *cysB, cysE, cysK, metA* and *metB* were overexpressed (*P* < 0.05) (**Figure 3B**). The occurrence of cysteine in the basal CDM_PRL2010_ (bCDM + Cys) does not seem to modulate expression of genes involved in sulfur amino acid metabolism. Glutathione (bCDM + Glut) does not allow a significant growth of PRL2010 (OD600 values of 0.31 ± 0.038). However, it enhanced the transcription of the *cysB*, *cysE*, *cysK, metA* and *metB* genes (*P* < 0.05).

Regarding complex substrates, *cys* genes appear to be less induced when PRL2010 cells are cultivated in whey protein compared to basal CDM_PRL2010_ in the presence of casein hydrolysate (bCDM + Whey and bCDM + Casein respectively in **Figure 3B**), although at significant level (*P* < 0.05). Moreover, casein hydrolysate significantly increases the transcription level of *metC*, the cystathionine β-liase (*P* < 0.05).

In all conditions tested, no significant transcriptional changes were detected for the *metC* and *metE* genes predicted to encode for cystathionine β-liase and homocysteine methyltransferase, respectively, except for *metC* when PRL2010 was grown in bCDM_PRL2010_ with casein hydrolysate.

## Discussion

Following birth, the human intestine is rapidly colonized by a vast array of microorganisms. *Bifidobacterium*, and in particular, *B. bifidum* strains are abundant in breast-fed infants, due to their capacity to grow on mucin and on human milk oligosaccharides (Turroni et al., 2010). The bifidogenic effect of breast milk due to bioactive peptides presence is well known (Liepke et al., 2002). A similar bifidogenic effect was shown for milk-derived k-caseins with loss of activity when the disulfide bonds were oxidized (Poch and Bezkorovainy, 1991).

Here, we investigated for the first time sulfur-containing amino acid metabolism in *B. bifidum* PRL2010 through the development of a CDM called CDM_PRL2010_ and by the molecular characterization of the putative auxotrophic behavior of this strain for cysteine. Data indicates that bCDM_PRL2010_ does not support *B. bifidum* PRL2010 growth, unless cysteine addition. The same behavior was extended to three other *B. bifidum* strains, i.e., LMG13200, LMG11582 and LMG11583. Furthermore, cysteine auxotrophy is not a common feature of all the (sub)species harboring the genus *Bifidobacterium*, since representatives of some species such as *B. boum, B. minimum, B. pullorum, B. ruminantium, B. saguini* and *B. scardovii* are able to grow without cysteine, although rather poorly.

*In silico* analyses of PRL2010 genome did not reveal the presence of the genetic arsenal needed to sulfate transport and reduction to sulfide. Growth experiments showed that cysteine is the only amino acid necessary to sustain PRL2010 growth but when the strain is cultivated in basal CDM_PRL2010_ with cysteine (bCDM + Cys) the transcription of genes involved in cysteine and methionine metabolism was not stimulated by the availability of these amino acid residues. Similar results were reported previously for other bacterial species, such as *Escherichia coli* (Kredich, 1992), *Bacillus subtilis* (Mansilla and de Mendoza, 2000), and *Lactococcus lactis* (Fernandez et al., 2002). Another reduced sulfur compound was used to understand if the role of cysteine in PRL2010 is linked to the reducing effect that it exploits on the redox potential (Even et al., 2006). However, reduced glutathione does not sustain any appreciable strain growth, yet enhanced the transcription of genes predicted to be involved in sulfur amino acid metabolism (*cysB*, *cysE, cysK, metA* and *metB)*. Similar behavior was previously reported for *E. coli* (Kredich, 1992) and *B. subtilis* (Mansilla and de Mendoza, 2000).

Complex substrates from dairy industry such as whey proteins and casein hydrolysate act as a reservoir of amino acid, peptides and free protein. Transcriptional analysis showed that whey proteins and casein hydrolysate increased the transcriptions of genes involved in serine degradation and/or conversion to cysteine and methionine (*cysB*, *cysE, cysK metA* and *metB*).

## Conclusion

This study provides new insights into the amino acid utilization ability of the *B. bifidum* species. This work also suggested the existence of a relationship between the sulfur amino acid metabolism and the redox state of the cell. The use of complex nitrogen sources available in the infant gut revealed an enhancement of growth yield and expression of genes involved in sulfur amino acid metabolism in PRL2010. These results could open a new avenue of research for the development of novel functional foods based on milk caseins and whey proteins with high content of cysteine or cysteine precursor’s compounds that could act as prebiotics for *Bifidobacterium* enrichment.

## Author Contributions

CF performed the work and wrote the manuscript, SD performed the work, CM, performed bioinformatics analyses, LM performed bioinformatics analyses, GL performed bioinformatics analyses, MM performed the work, AV performed the work, MO contributed data, DvS wrote the manuscript, MV wrote the manuscript.

## Conflict of Interest Statement

The Guest Associate Editor David Berry declares that despite having hosted a Frontiers Research Topic with the authors Marco Ventura and Francesca Turroni, the review process was handled objectively. The authors declare that the research was conducted in the absence of any commercial or financial relationships that could be construed as a potential conflict of interest.
