# Supplementary material for: Exploring Amino Acid Auxotrophy in Bifidobacterium bifidum PRL2010
Source: Front Microbiol. 2015 Nov 24;6:1331. doi: 10.3389/fmicb.2015.01331 (PMC4656816; doi:10.3389/fmicb.2015.01331)
Supplement: Supplementary file 1 [file Table_1.DOCX]

**Exploring amino acid auxotrophy and prototrophy in *Bifidobacterium bifidum* PRL2010**

**Supplementary Table**

**Table S1.** Blast results obtained for identification of genes involved in sulfur recovery from environment in *B. bifidum* PRL2010

| **Locus tag** | **Predicted function** | **Coverage %** | **ID %** | **Reference for BLAST search** |
| --- | --- | --- | --- | --- |
| BBPR_0202 | ABC transporter | 69 | 26 | Even et al., 2006 (*ssuD*) |
| BBPR_0362 | ABC transporter permease | 88 | 39 | Even et al., 2006 (*ssuB*) |
| BBPR_0324 | Sodium dicarboxylate symporter | 84 | 29 | Even et al., 2006 (*tcyP*) |
| BBPR_0668 | Glutamate transport ATP binding protein *gluQ* | 98 | 53 | Even et al., 2006 (*tcyC*) |
| BBPR_0671 | Glutamate transport system *gluD* | 78 | 26 | Even et al., 2006 (*tcyB*) |
